# State-wide implementation of patient-reported outcome measures (PROMs) in specialized outpatient palliative care teams (ELSAH): A mixed-methods evaluation and implications for their sustainable ﻿use

**DOI:** 10.1186/s12904-022-01109-w

**Published:** 2022-12-02

**Authors:** Hannah Seipp, Jörg Haasenritter, Michaela Hach, Dorothée Becker, Dania Schütze, Jennifer Engler, Stefan Bösner, Katrin Kuss

**Affiliations:** 1 Department of General Practice and Family Medicine, Philipps-University of Marburg, Karl-Von-Frisch-Straße 4, 35032 Marburg, Germany; 2 Professional Association of Specialized, Palliative Homecare in Hesse, Weihergasse 15, 65203 Wiesbaden, Germany; 3 Institute of General Practice, Goethe-University Frankfurt, Theodor-Stern-Kai 7, 60590 Frankfurt am Main, Germany

**Keywords:** Palliative care, Home care services, Quality of health care, Patient reported outcome measures, Routinely collected health data, Implementation science, Qualitative research, Surveys and questionnaires

## Abstract

**Background:**

Such patient-reported outcome measures (PROMs) and patient-centered outcome measures as the Integrated Palliative Care Outcome Scale (IPOS), Phase of Illness, and IPOS Views on Care (IPOS VoC), facilitate patient-centered care and help improve quality. To ensure sustainability, implementation and usage should be adapted according to setting. When settings involve several distinct teams that differ in terms of views and working practices, it is more difficult to integrate outcome measures into daily care. The ELSAH study aimed to learn how health professionals working in specialized outpatient palliative care (SOPC) viewed the use of these outcome measures in daily care, and what they express is needed for successful sustainable, state-wide application.

**Methods:**

We used a parallel mixed-methods design involving three focus groups (n = 14) and an online-survey based on normalization process theory (n = 76). Most participants were nurses and physicians from 19 SOPC-teams in Hesse, Germany. We used a triangulation protocol including convergence coding matrices to triangulate findings.

**Results:**

The majority of health professionals were able to integrate the outcome measures into their working lives and said that it had become a normal part of their day-to-day work. To ensure their sustainable integration into daily care, the motivation and concerns of health professionals should be taken into consideration. Health professionals must clearly recognize how the measures help improve daily care and quality evaluation.

**Conclusions:**

To implement the outcome measures in a number of teams, it will be necessary to take individual team characteristics into account, because they influence motivation and concerncs. Further, it will be necessary to offer opportunities for them to engage in peer support and share information with other teams. The sustainable use of outcome measures in SOPC will require continuous support within each team as well as across teams. When several distinct teams are working in the same setting, a cross-team coordination unit can help to coordinate their work efficiently.

**Trial registration:**

German Clinical Trials Register DRKS-ID: DRKS00012421; www.germanctr.de/DRKS00012421

**Supplementary Information:**

The online version contains supplementary material available at 10.1186/s12904-022-01109-w.

## Background

In palliative care, patient-reported outcome measures (PROMs), patient-centered outcome measures and caregiver-reported outcome measures enable patients’ and families’ needs to be recognized and addressed [1]. Evidence that their use improves patient-relevant outcomes is growing [2]. Further, their use allows quality of care on a provider level to be improved through case-by-case evaluation and by monitoring care on a policy level [3].

In palliative care, a variety of outcome measures exist. Some focus on specific domains e.g. on physical, social, spiritual or cultural domains [4]. Some consider special target groups like patient, relatives, health professionals or the healthcare system [5]. The European Association for Palliative Care (EAPC) recommends to use validated outcome measures, that include patients and relatives, and cover all relevant aspects of care [1]. Initiatives like the Australian Palliative Care Outcomes Collaboration (PCOC) and the Outcome Assessment and Complexity Collaborative (OACC) developed comprehensive approaches to evaluate palliative care [6, 7].

The implementation of outcome measures is complex and needs to be adapted to the setting in which it is used [8]. The Consolidated Framework for Implementation Research (CFIR) implies that successful implementation must take characteristics of the intervention, the implementation process, the individuals, as well as the inner and outer setting into account [9]. Implementation in a broad setting involving different healthcare professionals presents an additional challenge because of differing backgrounds, views, management and working practices. Nevertheless, a degree of standardization and guidance helps promote fidelity and improve data quality. This condition must be fulfilled if benchmarking and national and international care comparisons are to be possible [1].

Health professionals’ views must be taken into consideration if usage is to be sustainable. In a similar study, a negative attitude of health professionals to quality measurement reduced the chance of successful implementation [10]. Previous research has also shown that participants may refuse to use PROMs if they feel not to have enough time and training for use [11, 12]. Lind et al. found a general feeling of fatigue followed changes to routines in various palliative-care settings [13]. Implementation research has shown that if staff do not feel that a task is appropriate to their job responsibilities, rejection and reduced fidelity may result [14]. Implementation frameworks can help organize tasks and improve implementation [15]. Normalization Process Theory (NPT) focuses on the work that needs to be done to implement, embed, and sustain practices in different contexts. For this purpose, the theory considers the four core constructs coherence (how health professionals make sense of a new practice), cognitive participation (relational work to redesign the work), collective action (to sustain use), and reflexive monitoring (how the health professionals appraise the impact on work) [16]. This theory is therefore particularly suitable for studying the normalization of change in the work of teams, and considering the perspectives of involved health professionals. Therefore, NPT has been widely used in research to evaluate and understand implementation processes [17, 18].

Specialized outpatient palliative care (SOPC) provides comprehensive care to patients with life-limiting diseases and complex needs in their familiar surroundings [19]. The aim of our ELSAH study (‘Evaluation of Specialized Outpatient Palliative Care by taking the example of Hesse’) was to implement outcome measures in all SOPC-teams in Hesse, Germany. Before our study patient records were based on the German National Hospice and Palliative Care Registry and, although including data on structure and process quality, symptoms, treatment and support needs, they barely considered outcomes from the perspective of patients and relatives [20]. To find out more about day-to-day care and include a quality assessment from their perspective, we added patient-reported and caregiver-reported outcome measures [21].

In a first step, we examined key features of successful care from the perspective of patients, relatives and health professionals. We found that ‘treatment of complex symptoms, comprehensive care and a sense of security, as well as a focus on the quality of relationships, respect for individuality and the facilitation of self-determination’ [22] are relevant topics that need to be covered. On this basis, we assembled a set of validated outcome measures, which is based on the Outcome Assessment and Complexity Collaborative (OACC) suite of measures [6]. The set of outcome measures includes the Integrated Palliative care Outcome Scale (IPOS) [23], Phase of Illness [24], and IPOS Views on Care (IPOS VoC) [25]. In the next step, we examined the feasibility, acceptability and appropriateness of these outcome measures in a sample of five SOPC-teams. We found that they should be used considerately in order to minimize the burden on patients and relatives, and ensure their administration was manageable. It was also important that participants understood that the measures were useful [26].

However, it is not yet clear how the sustainable implementation of these outcome measures can take place in a broad SOPC setting involving different healthcare professionals, backgrounds, views, management and working practices. Because the health professionals are the ones who use the outcome measures, and their attitudes influence sustainable use, their perspective is decisive. Therefore, we evaluated the state-wide use of these outcome measures in daily care from the perspective of health professionals in this study.

## Methods

### Aim

We aimed to understand how health professionals working in SOPC viewed the use of outcome measures in daily care, and to determine what they express is needed for successful sustainable, state-wide application. We assumed that the implementation and application of outcome measures in daily care is a complex, dynnamic, and intersubjective process [27]. Therefore, we chose to use normalization process theory (NPT) as an explanatory framework in order to investigate embedding into routine care and to gain an insight into participants' experience of implementation [16].

### Design

We used a parallel mixed-method design. Althought we first conducted the qualitative focus groups and then a semi-structured online-survey, we analyzed them in parallel due to organisational reasons [28]. We considered mixed-methods appropriate and used an online survey to gather information on the views of professionals, and focus group discussions to understand participants’ views in more in depth [29]. We followed Mixed Methods Article Reporting Standards (MMARS) (supplemental material A) [30].

### Setting

We gradually implemented the outcome measures in daily care from November 2018 to June 2019 via the electronic documentation systems (EDS) of 19 SOPC-teams caring for adults in Hesse, Germany. In a further three teams, implementation could only take place after data collection due to delayed software adaptation. Hesse is a federal state in Germany containing rural and urban areas and about 6.3 million inhabitants [31]. In total, 22 SOPC-teams provide state-wide care for adults. Teams mainly include nurses and physicians, but in some cases also involve psychologists and social workers. All SOPC-teams belong to the Professional Association of Specialized Palliative Homecare in Hesse, which collects standardized data in order to improve quality of care [32].

We conducted a training meeting with every team on their premises before implementation. Topics were reasons for implementation, the content and practical use of the outcome measures, and organizational integration into daily care routines. After the training meeting the SOPC-team members applied the outcome measures in every case. Table 1 describes the outcome measures used. For the self-report, the SOPC-team members included the items into the conversations. Because we found in a previous study that it needs tact to address sensitive topics, we did not arrange predefined time points for self-report, but made the default to assess every topic when the information was collectible [26]. If a self-report was not possible, e.g. due to deteriorating health, the health professionals asked relatives or completed proxy-reports themselves.

**Table 1:** Outcome measures used

Outcome measure	Content	Target population	Application
Integrated Palliative care Outcome Scale (IPOS) [23]	Ten questions on the main problems, including physical, psychological, spiritual and practical concerns	Patients	Self-report (orally), proxy-report by relatives or HP (separately and/or alternatively)
Phase of Illness [24]	Five distinct phases of patients’ and relatives’ care needs, and the suitability of current care	Patients	Proxy-report by HP
IPOS Views on Care (IPOS VoC)—patients version [25, 33]	Four questions on the impact the SOPC-team had on patients’ quality of life and their main problems	Patients	Self-report (paper version handed out to patients; EDS entry after completion)
IPOS VoC: Question (no.4) on quality of life	One question on how patients rate their overall quality of life	Patients	Self-report (orally), proxy-report by relatives or HP (alternatively)
Views on Care (VoC)- relatives’ version^a^	Four questions on the burden on relatives and the impact the SOPC-team had had on their relatives	Relatives	Self-report (paper version handed out to relatives; EDS entry after completion)
VoC: Question on support provided for relatives	One question about whether the family caregiver felt the family was currently receiving enough help from their SOPC-team	Relatives	Self-report by relatives (orally), or HP (alternatively)

^a^developed as part of the ELSAH study based on IPOS Views on Care, unvalidated German version; HP = health professionals

### Data collection

#### Focus groups

Two experienced qualitative researchers (HS, KK) conducted focus groups to investigate SOPC-team members’ experiences and suggested changes. We developed the focus group topic guide by including the NPT core constructs (supplemental material B). We brought members from different teams together in order to stimulate a discussion [34]. We sent an email invitation to our contact partner in every team and asked them to forward it to team members and to choose one to three team members, independent of profession, to participate in a focus group. We did not sample for specific characteristics, but included the people who had interest to participate (convenience sampling) [35]. Participants gave their written informed consent to participate, permitted a video recording to be made, and provided demographic data in a questionnaire. We wrote field notes on the researchers’ thoughts directly afterwards.

#### Online survey

We used lime-survey [36] to conduct an anonymous semi-standardized online survey, based on the NoMAD (Normalization MeAsure Development) instrument [37, 38]. Together with other authors, the authors of the NPT, May and Finch, developed the NoMAD tool, which aims to understand participants’ implementation experiences. This validated generic quantitative measure contains 43 items and has been used in numerous previous studies [39–41]. It includes statements related to the core constructs of NPT and asks participants to rate their agreement on a 5-point Likert scale (strongly agree to strongly disagree plus the option ‘not relevant to my role’ and the possibility to omit questions) [37].

In line with the developer’s recommendations, we adapted NoMAD to suit our research interest by adding both an introduction and questions on participants’ characteristics [38]. We further added a question on how relevant they considered each outcome measure and gave them the opportunity to add comments in free text (supplemental material C). We emailed a participation link to all team leaders, and asked them to forward it to all team members involved in using the outcome measures (convenience sampling).

### Analysis

#### Focus groups

We transcribed and pseudonymized the audio recordings using MAXQDA [42]. May et al. presented several possibilities to analyze qualitative data in line with NPT [18]. We (HS, KK) used qualitative content analysis, and combined inductive and deductive coding using MAXQDA [43]. By setting the four core constructs of NPT (coherence, cognitive participation, collective action, reflexive monitoring) and their respective outcome measure as a-priori codes, we were able to link the content to the online-survey and identify issues associated with a specific measure. We discussed and added inductive codes and assigned them to the four core constructs of NPT after initial coding.

#### Online survey

As suggested by the authors of the NoMAD, we (HS, KK) used Microsoft Excel, version 2016, to analyze the online survey descriptively by looking at the response distribution and percentages [38]. We (HS, KK) used MAXQDA to analyze the free-text answers using qualitative content analysis [42]. We used the same coding tree, that we have elaborated for analysing the focus groups.

#### Mixed-Methods integration

We triangulated the findings of the focus groups and the survey on and interpretation level by using a triangulation protocol [44]. We created a convergence coding matrix for NPT core constructs and another convergence coding matrix for specific feedback on outcome measures. During the process, we examined the key quantitative and qualitative findings for convergences, dissonances, complementary information and silences, and developed an overarching conclusion for each component [44].

#### Deduction of topics requiring particular attention

In the analysis we identified topics, that reoccured in all NPT core constructs. We assumed that, from the participants' point of view, these issues need to be addressed if further use is to be successful. From this, we have deduced that these topics require particular attention for sustainable use of the outcome measures. Therefore, we present the deduction and the topics contents separately in this article.

## Results

### Study sample

We conducted three focus groups with n = 14 SOPC-team members. Five participants cancelled at short notice due to time constraints. Discussions took place in September and October 2019 in the rooms of an independent palliative association in Fulda and at the University of Frankfurt, and lasted about 105 min. The time between implementation and data collection was 3–10 months, depending on team.

The anonymous online-survey was conducted from November 20, to December 31, 2019. At this time the outcome measures had been used for six to twelve months. In the end, n = 76 complete data sets were available, and 104 hits were registered in total. Detailed results of the online-survey are presented in supplemental material C.

Participants’ characteristics are shown in Table 2.

**Table 2:** Characteristics of participants

		1^st^ focus group	2^nd^ focus group	3^rd^ focus group	Focus group participants in summary	Online survey participants
Number of participants; n		5	3	6	14	76
Duration; minutes		107	105	103	315	-
Gender; *n* (%)	Female	5	2	5	12	58 (76.3)
	Male	0	1	1	2	14 (18.4)
	n/a	-	-	-	-	4 (5.3)
Age; years	Mean (Min, Max)	51.2 (48, 57)	47.3 (45, 50)	57.7 (52, 68)	53.1 (45, 68)	49.5 (31, 69)
Profession; *n* (%)	Nurse	5	3	4	12	42 (55.3)
	Physician	0	0	2	2	24 (31.6)
	Administrator	0	0	0	0	6 (7.9)
	Other^a^	0	0	0	0	4 (5.3)
Work experience; years	Mean (Min, Max)	5.6 (2, 10)	10.3 (9, 12)	7.8 (5, 11)	7.6 (2, 12)	6.6 (1, 15)
Geographic location; *n* (%)	Urban	0	2	3	5	22 (29)
	Suburban	3	0	2	5	28 (36.8)
	Rural	2	1	1	4	26 (34.2)

n/a = not available; ^a^ The following information was provided under ‘Other^‘^: Team leader nursing (*n* = 3), Physiotherapist (*n* = 1)

In the following, we present the general appraisal and results according to NPT. Subsequently, we show key themes requiring particular attention, including specific findings on outcome measures.

### Appraisal of use in daily care

In the overall appraisal, about 69% of participants in the online-survey said the use of the outcome measures currently belonged to their day-to-day work. Participants that disagreed generally believed this would not be the case in the future (supplemental material C).

We present our findings according to the four core NPT constructs in Table 3. For each subject in the online-survey, we present a synopsis of online-survey results and focus group findings, provide a statement on the agreement between findings, and draw a conclusion. In Table 3, we also present a summary for each core construct of NPT.

**Table 3:** Convergence coding matrix for NPT core constructs

Subject	Synopsis of online-survey results^a^	Synopsis of focus group findings	Agreement between online-survey results and focus group findings	Conclusion
**Coherence:** ** → How do participants evaluate the informative value of the outcome measures?** ** → Is there a common understanding of the purpose behind them?**				
Differentiation between old and new documentation	82% agreed that they knew the difference, 3% disagreed (12% neither)	Differences are known, but sometimes confusion occurs as to which changes our study is responsible for	Convergence	Participants appear to be aware of the differences between the old and new documentation
Shared understanding of the aim/purpose in the team	43% considered a shared understanding to exist, 37% did not (12% neither)	Some participants said they use the documentation because they are required to. Others thought the items were of value in their daily work	Convergence	There does not seem to be a shared understanding of the aim/purpose
Potential value in practice	37% considered the outcome measures to provide added value in their work, 42% do not (20% neither)	Some participants valued changes such as new response options and new items. Others considered the previous documentation to be sufficient	Complementarity (which changes participants welcomed and comparison to previous documentation)	Several participants saw no added value in practice. This may be because they considered the previous documentation to have been sufficient
Identification with the topics	57% could identify with the topics, 30% could not (9% neither)	Most participants considered the topics to be relevant to care. There was more discussion about whether the outcome measures captured the required information	Complementarity (concerns related more to measures than issues)	Most participants identified with the topics. Those that said they could not seemed to struggle with the measures rather than the topics
Modified documentation revealed changes in quality	26% believed the quality of care could be evaluated this way, 53% did not (18% neither)	Some participants generally doubted that quality can be revealed in documentation, and said quality could only be seen in practical interaction. Most participants disagreed that outcome measures could depict reality but could imagine that outcome measures might provide an indication of care quality	Complementarity (doubt on principle, but not necessarily related to the measures used here)	The majority of participants doubted that quality could be revealed through the use of the documentation. Participants seemed to doubt whether outcome measures can provide a real representation of care, rather than want to criticize the employed measures
Concern about negative impact on specialized outpatient palliative care	43% were concerned, 24% were not (25% neither)	Some participants feared their data might be misinterpreted and lead to a poor assessment of their work, whether or not they did a good job in practice. They were also concerned that time taken for documentation took time away from patients	Complementarity (what concerns participants have)	Several participants feared such documentation might have a negative impact on specialized palliative home-care. They feared the documentation of poor health in their patients might have a negative impact on the evaluation of their work and result in less time for patient care
Time for documentation is invested wisely in quality of care/patient care	30% agreed that the time was invested wisely, 46% disagreed (21% neither)	Some participants thought time spent with the patient was particularly valuable and important for the relationship with him or her. For participants that saw no added value for practical care, documentation was simply extra work invested for external evaluation. Spending a lot of time on such assessments seemed to them to be counterproductive	Complementarity (explanation of why time is perceived as not being invested wisely)	Several participants did not feel that time taken for documentation was well spent in terms of quality of care. This seemed to be particularly true when they saw no added value for their practical work. For them, spending time on assessments seemed to be counterproductive, i.e. lead rather to a deterioration than an improvement in care
**Summary on coherence:** It became apparent that not all participants shared a common understanding of the purpose of the measures. Although participants could identify with the topics, they doubted whether documentation would benefit their practical work and feared poor evaluations and a negative impact on practical work. Coherence is limited by general doubts about whether quality can be depicted through the use of outcome measures				
**Cognitive participation:** ** → How do users engage in the use of the outcome measures?** ** → How do users develop a collaborative approach to using them?**				
Key people in the team encouraged the continuous use of the outcome measures	62% agreed that some people in the team encouraged use of the measures, 8% disagreed (16% neither)	All participants said that at least one person in their team was responsible for documentation and encouraged its implementation and continuous use. They generally also made up the focus groups	Convergence	In most teams, at least one person encouraged continuous use of the modified documentation
Key people outside the team encouraged their use	42% agreed that people outside the team encouraged their use, 18% disagreed (16% neither)	All participants described the staff of the Professional Association of Specialized Palliative Homecare in Hesse as key people outside the team that encouraged its use Some participants also named our study team as among the people encouraging the use of the modified documentation	Complementarity (who are the key people)	Some participants agreed that key people outside the team encouraged its use. The Professional Association of Specialized Palliative Homecare in Hesse and the study team were among those named
Knew how the documentation should ideally be used	83% agreed to knowing how the documentation should ideally be used 7% disagreed (5% neither)	Participants were familiar with the new documentation. However, it became obvious in discussions that details concerning its practical use and background were not completely clear. For example, they felt unsure how to address psychosocial questions	Complementarity (where insecurities prevail)	Although most participants felt they knew how the documentation should ideally be used, it became obvious that details were unclear
Theoretical examination of topics	86% agreed that they had dealt with the topics theoretically (5% neither, 4% disagreed)	Participants knew what had changed in the modified documentation, and most participants agreed that added topics played an important role in their work	Convergence	Most participants said they have examined the additional topics in the modified documentation
Motivation to use the documentation in practice	41% were motivated to use the documentation in practice, 38% were not (16% neither)	Participants explained that the overall workload linked to the documentation reduced their motivation. They felt that the modified documentation created additional work for health professionals because it contained more items. Participants also said team members became frustrated when it did not work as they had hoped in practice. The sharing of information among teams and team-related feedback were described as encouraging use	Complementarity (which factors influence motivation)	About the same number of participants agreed as disagreed to being motivated to use the documentation with patients. The overall burden of documentation as well as problems in its practical use may have reduced motivation. The sharing of information among teams and team-related feedback may increase motivation
Support for the modified documentation	29% agreed to support the documentation and 41% did not (25% neither)	Participants that expected use of the documentation to result in improvements were more likely to support and try to integrate it. Those that expected no improvements tended not to support its use and to want to undo the changes	Complementarity (influence of assumed benefit)	Participants were divided on support for the modified documentation. Those that expected benefits from it tended to support it and those that did not tended not to
**Summary on cognitive participation:** Support for cognitive participation varied. Key persons that encouraged use of the revised documentation and expected benefits from it supported a collaborative approach to using it. The assumed benefit is influenced by participants’ theoretical understanding and perceived feasibility. Motivation for use was reduced by the overall burden of documentation and promoted by information sharing across teams and team-related feedback				
**Collective action:** ** → Is collective action employed to promote the use of the outcome measures?** ** → What factors hinder or promote the use of the outcome measures in day-to-day work?**				
Easy integration into day-to-day work	While 42% said they could integrate the modified documentation into their day-to-day work, 32% said they could not (18% undecided)	Participants described symptom documentation as easy to use. They explained that the documentation on psychosocial issues and the assessment of self- and proxy-reports caused problems. Further, the organizational integration of paper-based IPOS VoC in day-to-day care was regarded as difficult. Some teams developed internal standards to facilitate use of the documentation, e.g. on how often to use the modified documentation in order to create a routine. Others tried to facilitate use of the documentation by sending out assessment documents by mail or conducting them on the telephone	Complementarity (which measures caused problems, initiatives to improve these)	Several participants had problems integrating the modified documentation into their day-to-day work. They described problems with documentation relating to psychosocial issues, with self- and proxy assessments, and with the integration of the paper-based IPOS VoC. Some teams developed internal strategies to facilitate use of the modified documentation
Improvements in cooperation with team colleagues	18% agreed, but 50% disagreed that the modified documentation had led to improvement in cooperation with colleagues (26% neither agreed nor disagreed)	The participants described different ways of using the documentation in their teams. Some discussed the results in team meetings, and those that did particularly appreciated IPOS Phase of Illness and IPOS. Others did not discuss results with colleagues, but at least looked at the documentation before visiting patients. They explained that from their point of view the previous documentation had sufficed. Furthermore, they said information on patients was generally communicated orally rather than via documentation	Complementarity (the way the documentation was used in day-to-day care influenced whether advantages were seen)	Several participants disagreed that the modified documentation led to improvements in cooperation with colleagues. The use of the documentation in day-to-day care apparently differed, with those that used it appreciating IPOS Phase of Illness and IPOS for its impact on cooperation with colleagues. Those that did not already use the documentation in team meetings may have seen fewer advantages in terms of cooperation with colleagues. This may be why a minority recognized a positive impact on cooperation with colleagues
Improvements in the quality of relationships with patients and relatives	13% agreed, but 48% disagreed that the modified documentation improved the quality of relationships (28% neither agreed not disagreed)	Although most participants agreed that the topics are important, many emphasized that relationships are built during interactions with patients rather than via documentation. Some participants feared that the strict use of documentation might damage relationships and stressed that a sense of balance was necessary, especially with respect to IPOS psychosocial questions	Complementarity (Reasons why participants think the quality of relationships might suffer)	Only a few participants agreed that use of the modified documentation improves the quality of relationships. Reasons for this may have been that the participants considered relationship quality to be influenced more by interacting with patients than by documentation. Furthermore, they feared that the relationship could be damaged by strict application of documentation
Everyone in the team can use it according to instructions	71% agreed that everyone in the team could use the modified documentation, 18% disagreed (7% neither)	As use of the documentation is obligatory for all participants, they used it in practice and knew how to do so. Small misunderstandings and uncertainties became clear, e.g. on frequency of use	Convergence	Most participants agreed that everyone in the team could use the modified documentation in accordance with instructions
Sufficient support offered during implementation phase	63% agreed that support was sufficient during the implementation phase, 20% disagreed (9% were undecided)	Participants were pleased with the implementation and all teams commented favorably on the personal training and the opportunity to ask questions. Team leaders felt that explanations provided by the study team were better received than their own	Convergence	Most participants agreed that the support offered during the implementation phase was sufficient
Sufficient support is provided	60% agreed that sufficient support was provided at the time of the survey, 16% disagreed (17% were undecided)	Some participants wished for continuous support from the experts in the use of outcome measures, because they sometimes felt unsure about details or technical issues relating to the EDS	Dissonance	While most participants in the online-survey agreed that support was sufficient at the time of the survey, participants in the focus groups would have welcomed more continuous and more regular support from experts in the use of the outcome measures. This may be because several focus group participants were members of the management team and were contacted by colleagues when questions arose. They may therefore have perceived a greater need for support
Sufficient resources are available for use in everyday care	While 40% agreed, 30% disagreed that sufficient resources were available for use in everyday care (22% neither agreed nor disagreed)	Some participants said sufficient resources were available. Others felt that they did not have sufficient time. These participants emphasized the overall burden of documentation and that it had increased as a result of additional items in the modified documentation. Some participants described attempts to counter this by delegating organizational issues to one team member	Complementarity (Resources mainly related to time, strategies for improvement)	Participants were divided over whether sufficient resources are available for use in everyday care. Those who felt they did not have sufficient resources said they lacked time for documentation
The management team provides sufficient support	72% agreed that the management team provided sufficient support for use of the modified documentation, 7% disagreed (11% neither)	Some management team attended the focus groups, so the adequacy of their support was not discussed. They themselves said it was difficult for them to motivate their teams over time	Silence	Most participants agreed that the management team provided sufficient support in the use of the modified documentation. Members of the management team said they had problems motivating their teams over time
**Summary on collective action:** Obstacles to collective action were the difficulty of integrating the documentation into day-to-day work and concerns about lessening the quality of relationships. The way the documentation was employed in day-to-day care had an influence on whether it was seen to be beneficial. Participants were divided as to whether enough support was provided and enough resources were available, but they agreed that their management teams provided sufficient support				
**Reflexive monitoring:** ** → How do the participants view the outcome measures?** ** → What improvements did they suggest?**				
Awareness of reports / experiences of usefulness	26% agreed they knew of reports/experiences of usefulness, but 43% disagreed (24% neither)	Most participants said they did not know of any such reports, and were uncertain how the data was evaluated. Some were afraid that poor final values would be considered as signs of poor quality	Convergence	Most participants were not aware of reports/experiences of the usefulness of the modified documentation
Benefit for their own work	24% agreed, but 47% disagreed to seeing any benefit for their own work (24% neither)	With respect to the advantages of using the modified documentation, participants distinguished between their practical work and quality evaluation In their practical work, participants valued the IPOS Phase of Illness, the symptom items of IPOS, the five-point Likert scale and the response option ‘not assessable’. Some participants did not see any improvement over the previous documentation. Some participants suggested that a clearer presentation in the electronic documentation system would help in their practical work Some participants did not understand how the documentation could help them evaluate their own work. They doubted whether an assessment could really be objective and said the proxy assessment might not be valid because of differing opinions among staff members. They also criticized patient self-assessments on the grounds that their condition could influence their assessment	Complementarity (differentiation between advantages in their practical work and in its use for evaluation purposes)	Participants were divided over the advantages of using the documentation in their own work. With respect to any advantages, they distinguished between their practical work and quality evaluation. Participants basically saw how their day-to-day care benefited but thought some items were difficult to integrate. They said it was difficult for them to understand how outcome measures could be used in quality evaluation
Fellow employees’ agreement on usefulness	13% agreed, but 51% disagreed that their fellow employees thought the modified documentation was useful (22% neither)	Agreement among their fellow employees was not discussed in the focus groups	Silence	Most participants disagreed that their fellow employees thought the modified documentation was useful
Confidence that feedback will improve	59% said they were confident that feedback would improve the modified documentation, 14% disagreed (21% neither)	Participants emphasized that their motivation might deteriorate if the teams’ feedback was not taken seriously. They trusted that their feedback would lead to improvement	Convergence	Most participants were confident that their feedback would improve the usefulness of the modified documentation
Ability to adapt use of the modified documentation to suit their own way of working	61% could adapt the application of the documentation to suit their own way of working, 17% disagreed (13% neither)	All participants knew it was possible to include the items in conversation. They reported, however, that the practical implementation was sometimes difficult. Furthermore, IPOS VoC was described as difficult to adapt to suit their own work methods	Complementarity (aspects causing problems adapting)	Most participants agreed they could adapt use of the modified documentation to suit their own work methods Integration into conversations and use of IPOS VoC were more difficult
**Summary on reflexive monitoring** Participants were divided over the benefits of using the documentation. They reported that it was basically useful in day-to-day care, but said some items were difficult to integrate. It was difficult for them to understand how outcome measures can be used in quality evaluation				

^a^values rounded; response options ‘not relevant to my role’ and ‘no answer’ not reported; response options ‘strongly agree’ and ‘agree’, as well as ‘disagree’ and ‘strongly disagree’ were combined to form one response

### Topics requiring particular attention

**Fig. 1:**
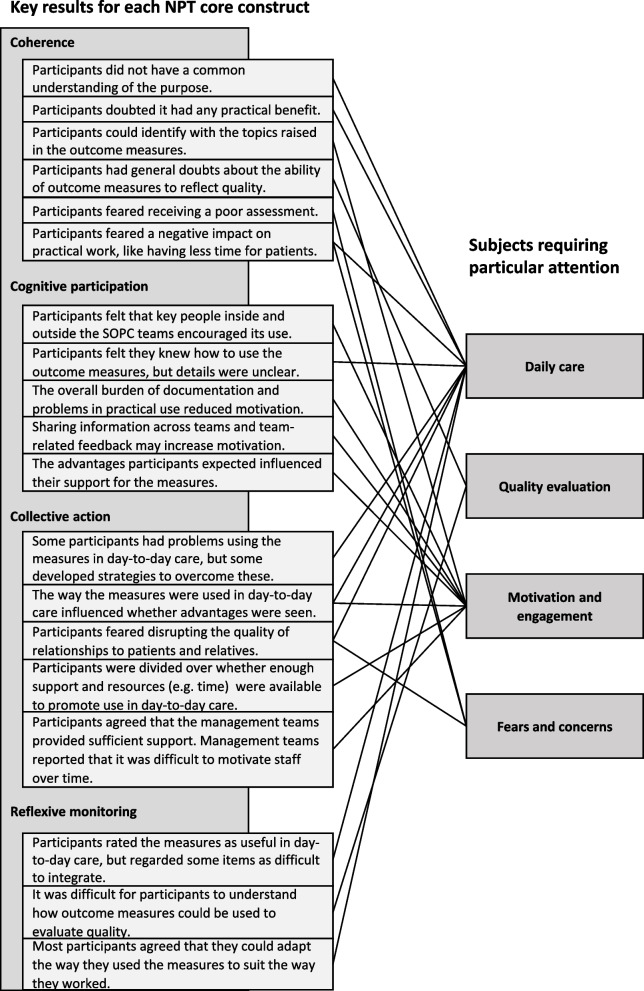
NPT core constructs leading to four key themes

We found that four key topics recurred across all NPT constructs (Fig. 1). From the perspective of health professionals in SOPC these topics require particular attention if the revised documentation is to be used sustainably in daily care: 1) Daily care, 2) quality evaluation, 3) motivation and engagement, and 4) fears and concerns. We illustrate our findings using translations of pseudonymized quotations from the focus groups.

#### Daily care

Most participants knew the difference between the old and new documentation, but several participants (42%) did not see that the new documentation provided additional value in their practical work. Correspondingly, many participants (46%) did not feel the time for documentation was well spent.

*The patient has always been taken into account to a great degree, but just not in the documentation. Now the documentation is written as if the patient really said all this, but everything is actually the same as before. (1738E, female nurse)*

Most participants (71%) assumed, that everyone in their team could use the documentation in day-to-day care, but we found they had difficulties integrating it. Participants that felt they did not have sufficient resources at their disposal thought they had too little time for documentation.

Many participants (50%) thought the use of the outcome measures promoted cooperation with their fellow employees. While some mentioned the results in their team meetings, others did not look at them after entering the data. It became clear that not all participants knew how to integrate the results into their work, which may explain a lack of appreciation of their usefulness.

#### Quality evaluation

Many participants (53%) doubted whether quality could be evaluated through the use of documentation. This reflected a general concern whether outcome measures accurately reflected the actual care situation:

*What I want to make comprehensible is that I can’t use a table to illustrate the way a person is, the emotions a person has, the process of dying in a person, or how a person deals with an illness. (1738E, female nurse)*

Some participants struggled with the idea of an objective assessment. They criticized proxy assessments as having no validity because the assessment of health professionals can differ. They also criticized patient self-assessments on the grounds that patients’ conditions might influence their assessment.

*But isn't it again a bit of a subjective judgment that varies from one colleague to another, when we go through the symptoms and I assess something? [...] So I look-, and of course ask about pain, but of course I assess what he tells me in terms of the different categories. My colleague might see things quite differently. How coherent is the picture then? 1736E, female nurse)*

It became obvious that most participants (57%) could identify with the topics covered by the measures. However, many participants (43%) had no knowledge of reports or experiences of the usefulness of such measures, which may explain why they were sceptical about their usefulness.

#### Motivation and engagement

Only 41% of participants were keen to use the outcome measures in practice. Beyond the overall burden of documentation, the assumed benefit also influenced their engagement. Those that assumed care would benefit showed more interest in using it than those that did not.

Uncertainty in using the documentation in practice may also have had a negative impact on motivation. Most participants had dealt with the topics theoretically (86%) and felt they knew how the outcome measures should ideally be used (83%), but the discussions showed that some details were unclear. For example, they were particularly unsure how to address IPOS questions on psychosocial issues.

*Yes, because how do I interpret the question, right? ‘At peace with myself’. Hmm. I have cancer, shit, I'm going to die. How can you be at peace with yourself ... That's just an expression covering one, two questions, or three, four (laughs), that's actually something everybody has a hard time with, all of us. (1742E, female nurse)*

We found that sharing information across teams and team-related feedback helped motivate them. Most online-survey participants agreed that sufficient support was provided during implementation (63%), and at the time of the online-survey (60%), and they agreed that the management team provided sufficient support (72%). Nevertheless, participants in the focus groups wished for more continuous training and regular support from experts in the use of outcome measures.

#### Fears and concerns

Some participants (43%) expressed concern about a negative impact on care. They were concerned they would have less time for patients because they would have to invest time in completing the documentation. They also feared data might be misinterpreted, and that their work would be poorly evaluated, even though they were doing a good job in practice:

*The Phase of Illness assessment, that's where the ‘stable’ bothers me. [...] But the thing is always: is he stable BECAUSE he has SOPC? [...] Stable actually means: does he still have an indication for SOPC? [...] And that’s when I ask myself: will this be used against us at some point? If we use that in the evaluation for the [health] insurers, for the funding agencies, will that be used against us? [...] Is that stable at some point the time they say ‘stable, stable, and you’re providing SOPC!’? (1745E, female physician)*

Several participants (48%) denied that the use increased the quality of relationships with patients and relatives. Participants reckoned the quality of relationships depended more on personal interactions than outcome measurements. They also feared that the quality of relationships might deteriorate because patients might find some of the items upsetting:

*But I find it very difficult to ask such questions because I’m then rather afraid that they might close themselves off completely. They are at home; we are already intruding on their intimacy, and so I think we should show a great deal of tact. (1744E, female nurse)*

### Specific feedback on the outcome measures

Participants regarded IPOS, and Phase of Illness symptom documentation as most helpful in their day-to-day care. They struggled with the psychosocial questions asked in IPOS (anxiety, family anxiety, depression, feeling at peace, share feelings). Although most participants considered the content to be relevant to care, several participants found it difficult to integrate the questions into a conversation, and to classify the answers on the scale. Participants said it was often impossible to interpret answers to such questions at the beginning of care because trust first had to be built up before such topics could be addressed. These issues could therefore not be raised when care was only provided for a short period.

Some participants criticized the wording of the questions. They also said it was difficult for staff members to make proxy assessments and almost impossible with cognitively impaired patients. Most participants appreciated the usefulness of IPOS VoC in evaluating care, but not in their practical work. Detailed feedback on the outcome measures is presented in Table 4.

**Table 4:** Convergence coding matrix regarding specific feedback on the outcome measures

Item	Quantitative results: online-survey results^a^			Qualitative findings: synopsis focus group findings and online-survey free-text response	Agreement between quantitative results and qualitative findings	Conclusion
	Mean (SD)	Median	Number of free text responses n (%)			
IPOS						
Main problems or concerns	7.1 (3.1)	8	10 (13.2)	Only a few participants commented on the main problems. They saw no problems in the responses and only suggested improvements in technical integration	Convergence	Participants regarded the main problems as relevant. The application did not cause problems in daily care
Symptom assessment (new: ‘how it has affected’ as opposed to ‘severity of symptoms’)	6.6 (3.1)	7	8 (10.5)	Participants rated symptom assessment as practicable and useful in their practical work. They said it was usually possible to ask about symptoms during a conversation, but that cognitive impairment complicated both the patient’s self-report and proxy-assessments. Participants took into account whether their own assessment was consistent with the patient’s self-report and considered this to be relevant to care. For the same reason, they welcomed the graphical representation of symptom trajectories Most participants welcomed the new questions asking about impairment caused by symptoms rather than about symptom severity. All participants appreciated the option to provide the response ‘not assessable’ when it was not possible to complete an assessment Participants were also concerned that symptoms often increased over the course of care and that this could be considered to reflect poor quality of care. Several participants were unsure how to deal with the final assessment because they were rarely present when patients were dying. They felt that both the assessment of the last contact and documentation after death were both biased. Most teams resorted to a default position of either documenting symptoms after every contact or at least once a week	Complementarity (Insecurity and concerns related to quality evaluation)	Participants considered symptom assessment to be relevant and useful in their practical work Some insecurity and concerns emerged with respect to quality evaluation
Anxiety	4.4 (3.4)	5	16 (21.1)	Most participants agreed that this is an important issue in palliative care. Several participants did not think it should be asked this way, but should be addressed more tactfully Some participants considered the question unnecessary because they regarded it as normal to be afraid when receiving palliative care. Others valued the item because the question gave them the opportunity to start a conversation on the topic	Disagreement	While the relevance is rated as rather low in the qualitative results, qualitative findings showed that some consider the topic to be relevant but to be difficult to raise in practice
Family anxiety	4.5 (3.3)	5	14 (18.4)	Most participants agreed that this is an important issue in palliative care. Several participants did not think it should be asked this way, but should be addressed more tactfully Some participants considered the question unnecessary because they regarded it as normal that the family should be afraid when a relative is receiving palliative care	Disagreement	While the relevance is rated as rather low in the qualitative results, qualitative findings showed that some consider the topic to be relevant but to be difficult to raise in practice
Depression	4.7 (3.5)	5	12( 15.8)	Most participants agreed that this is an important issue in palliative care. Several participants did not agree think it should be asked this way, but should be addressed more tactfully Some participants criticized the way the question was formulated	Disagreement	While the relevance is rated as rather low in the qualitative results, qualitative findings showed that some consider the topic to be relevant but to be difficult to raise in practice
Feeling at peace	3.1 (3.2)	2	28 (36.8)	Many participants were upset by this question. They agreed that it is an important issue in palliative care, but did not agree that the question can be presented in this way Some participants rejected the idea that patients receiving palliative care could be at peace at all. Others feared the topic might upset patients Some participants said they integrated the subject into conversations, and hardly any participant asked this question as it was presented in the documentation. They also said that when the subject was raised, it often provoked questions as to what was meant by the term. They emphasized that this question requires a high level of trust, as they were concerned that the quality of the relationship with the patient would otherwise suffer. For these reasons, participants felt uncomfortable in asking this question	Disagreement	While the relevance is rated as rather low in the qualitative results, qualitative findings showed that some consider the topic to be relevant but to be difficult to raise in practice
Share feelings	3.6 (3.3)	3	11 (14.5)	Several participants did not think the question should be asked this way, but thought the subject should be addressed more tactfully. They also emphasized that not some patients did not wish to discuss this issue	Convergence	Most participants felt this topic had no relevance to care
Information needs	5.2 (3.5)	5	7 (9.2)	Some participants reported that this item sometimes raises new questions. Some explained that it is one of their tasks to provide information and that the question should therefore not be included in the patient’s assessment	Convergence	Most participants felt this topic had no relevance to care
Practical problems	5.5 (3.4)	5	9 (11.8)	Most participants agreed that this is an important issue in palliative care. They said that practical problems should be discussed in great detail Some participants reported that affected patients often made unrealistic demands (e.g. desire for a night watch) and feared that this would have a negative impact on the evaluation	Convergence	Most participants felt this topic had no relevance to care
IPOS Phase of Illness						
IPOS Phase of Illness	7 (2.9)	8	8 (10.5)	Most participants appreciated the use of IPOS Phase of Illness and reported that they used it when communicating within their teams. They appreciated the fact that it included the complexity of care in the quality evaluation Some participants feared the description of a phase as ‘stable’ might be misinterpreted. They feared it could be understood to mean the patient should no longer be eligible for specialized care, rather than that the patient is stable as a result of specialized palliative home-care	Convergence	Most participants considered this item to be relevant. Some participants feared misinterpretation
IPOS Views on Care						
Question on quality of life	6 (3.4)	6	12 (15.8)	Most participants considered this topic to be relevant. Some participants described this question as very intimate and criticized the way it was formulated Some participants felt the question was inappropriate because they assumed that the quality of life of palliative patients is inevitably poor. They reported individual cases in which patients expressed their dislike of the question. Some participants described the scale of 1–7 as too broad Others found it straightforward to ask this question or to address it in a conversation	Convergence	Most participants considered this item to be relevant. Some participants feared misinterpretation
Question on the relatives’ support	5.5 (3.4)	6	10 (13.2)	Most participants considered this topic to be relevant. They said they asked the question regularly, and weaved it into their conversations. Some noted that the question could only be asked while care was being provided, although it was sometimes also relevant after death	Convergence	Most participants considered this item to be relevant. Some participants feared misinterpretation
Patient’s version as a whole	5.2 (3.6)	5	9 (11.8)	Most participants suspected it might be useful for evaluating quality, but only a few said they considered the results in their practical work Most participants feared that patients would not provide honest answers because of their dependence on the team. They therefore proposed integrating the questionnaire in an anonymous survey Participants reported that the questions were sometimes difficult for patients to understand because of the wording, so they sometimes filled out the questionnaires with the patients or presented the items orally Participants reported that it was a major effort for them to hand out the written questionnaires and collect them later	Complementarity (Different aspects within the relevance assessment)	Most participants considered the question relevant to quality evaluation, but did not use it in their practical work. They further reported difficulties approaching the subject
Relative’s version as a whole	5.1 (3.5)	5	8 (10.5)	As in the case of the IPOS patient’s version, participants criticized the effort involved and the lack of any usefulness of the responses in their practical work. However, they thought it would help in the evaluation of quality. They also mentioned the problem of patients’ passing away before the questionnaires had been collected, making them unavailable for evaluation	Complementarity (Different aspects within the relevance assessment)	Most participants considered the question relevant to quality evaluation, but did not use it in their practical work. They further reported difficulties approaching the subject

^a^ Question: ‘How relevant to your work do you rate each of the questions/topics in the new documentation?’; response options for all questions: scale 0 (not at all)—10 (completely)); range 0–10; additional option to answer in free text

## Discussion

### Main findings

Overall, participants said that they had accepted the use of the outcome measures in daily care and that it had become part of their day-to-day work. However, participants expressed that sustainable integration into daily care will require that special attention is drawn to their usefulness in daily care, in quality evaluations, in motivating and engagement and appreciating the concerns of health professionals.

### Comparison of findings with those reported in the literature

Feedback from participants in our study was heterogeneous, suggesting that the effectiveness of the implementation varied. While implementation worked well in some teams, this was not the case in other teams, suggesting that it is more difficult to implement in different teams than in individual teams. Top-down implementation might reduce the sense of accountability and possibly even lead to resistance when compared to bottom-up implementation [15]. Although the implementation by our research team was carried out in consultation with the Professional Association of Specialized Palliative Homecare in Hesse and thus with all SOPC-teams, it can be assumed that individual health professionals did not feel they had been included in the decision-making process.

In addition to the initial implementation, we found that the way the measures are used by the teams in daily care influences sustainability. Other studies found that supportive leadership and peer support facilitated the implementation and encouraged its further use [45, 46]. Bradshaw et al. said that the benefits need to be demonstrated in order to increase motivation and engagement and to promote and sustain a collaborative effort, adding that measures may otherwise be dismissed as pointless [47]. Another study agreed that good training and guidance is required in the use of PROM in care [48]. It became clear that training before and during implementation is not enough, but that continuous support, which should be adapted to the needs of individual teams, should be provided to users.

Sustainable motivation requires that results are interpreted and fed back to health professionals [46]. Our participants distinguished between benefits in their practical work and benefits in terms of quality assurance, but for its sustainable use it is important to understand both aspects. Use in daily care, e.g. in team discussions, may clarify the direct benefits in practical work and for individual patients. Similar to our findings, another study showed that health professionals appreciated the opportunity to recognize unmet needs through the electronic use of PROMs, but were also concerned that it could disrupt care delivery processes, especially when intimate questions were asked of patients with whom the duration of contact had been only short [49]. Other research, however, showed that psychosocial support is also possible on first contact [50]. On specific outcome measures, further feedback from our participants was in line with the results of similar studies [47, 51]. This shows the need for ongoing training, particularly on how to integrate the psychosocial questions of IPOS into daily care. Peers with experience of using the measures could regularly address issues surrounding integration and other problems in the team. By ensuring they were accessible to colleagues as low-threshold contact persons, they could also help train new staff. Cross-team information sharing amongst peers could improve the promotion of long-term, sustainable use. Implementation in several teams thus also offers opportunities for mutual support.

To enhance a good quality of nursing documentation, Groot et al. recommended to use familiar terms [52]. Following this recommendation, we used the term documentation in conversations with SOPC-team members about the outcome measures. Looking back, we realized that the term has negative associations and often worries health professionals because they do not see documentation as a supportive component of care, but rather as something separate. In further implementations, therefore other terms like patient- and care-relevant outcomes should be used, in order to underline the relevance for care and the patients and relatives and to strenghten the motivation for use.

We found concerns about negative consequences to be fundamental but related more to the health care system than to the outcome measures. For example, although the participants of our study considered the content of the outcome measure ‘Phase of Illness’ important, they also saw it as a potential threat. They were afraid the health care system might force them to exclude patients from SOPC, if the measure ‘Phase of Illness’ was recorded as stable. This critical attitude may result from our participants having dealt with quality assessment for some time, as this may have given them the impression that control by a higher authority (the health system), as reflected in more and more assessments, is increasing. Krawczyk et al. stated that health professionals rather focus on the micro- and meso-level in daily care, so the macro perspective needs to be explained to them [53]. Although this might reduce scepticism, individual teams may not have the resources to do this. A community of several teams could concentrate resources on key events and address these issues together. Further, internal benchmarking of teams within their community could enable best practices and areas of improvement to be identified, without producing fears of control [54].

### Strengths and limitations

The use of normalization process theory helped us focus on the relevant aspects of integration into daily care. The mixed-methods design offered the opportunity to gain a multi-perspective insight [38]. If we had used a sequential design with the online survey first, we could have considered the results in the focus groups, but this was not possible for organizational reasons. However, the online-survey enabled low-threshold participation, and anonymous participation may have made it easier for participants to make critical statements. The focus groups allowed a deeper understanding of the participants’ views.

For data protection purposes, we sent the invitations to participate to the team leaders and asked them to forward them to team members. Gatekeeping by the team leaders may therefore have influenced the results. Some participants could not participate because of their work obligations, or excessively long journeys to focus groups. In our focus group just two physiscians participated, but in total an adequate number of physicians could be recruited, so that the physician’s perspective could also be integrated. Furthermore, we did not include patients in this evaluation phase. However, it became apparent that team members were strongly patient-oriented and tried to call attention to their needs.

### Implications

We identified certain factors that may help promote the sustainable integration of PROMs and caregiver reported outcome measures in daily care. In addition to findings relating to the use of specific outcome measures, we also present measures that collaborating teams should use collectively. These results are probably transferable to similar home-care settings that are responsible for caring for adults. The transfer of results to the care of children and adolescents, or to inpatient palliative care, may be limited due to the differing needs of such patients and the different structure of their daily care [55]. We could only derive implications for a sustainable application from the feedback of SOPC-team members after a short period of application. Further research should focus on applying and evaluating strategies for sustainability and integrate the perspectives of patients and relatives.

The use of PROM as a basis for funding and accreditation has raised fears among our participants. They feared that, for example, higher compensation could be paid if the outcome measures were rated well and deductions could be made if they were rated badly. This should be carried out with caution, as stakeholders have differing interests and there is no clear evidence for its appropriateness in this regard [53]. However, we recommend internal use for comparisons with other teams and for internal quality assurance. Further research is needed on how results should be fed back to health professionals.

It became obvious that not all participants felt that the use of the outcome measures had or should become part of their day-to-day work. Previous research has shown that the relationship between implementers and users influences the success of an implementation. It may be helpful for similar implementation projects to make peers responsible for implementation, as their expertise may be better accepted than that of external staff [56]. Furthermore, it became clear that the measures should only be implemented over the long term, as the process is a continuous one that requires constant support and further development, along with appropriate resources.

## Conclusion

The sustainable integration of outcome measures into daily care will require that particular attention is paid to responding to the motivation and concerns of health professionals and making them aware of the value of the measures in daily care and quality evaluation. Combining the implementation of PROMs in a number of SOPC-teams is more complicated than implementation in a single team because teams’ specific characteristics must be taken into account and a comparable basis created. At the same time, combined implementation offers opportunities for mutual support and the pooling of resources. The provision of support by experienced peers within and across teams will enable concerns to be addressed and benefits in daily care to be explained on a low-threshold level. Centrally organized information sharing across teams is a suitable means of communicating the benefits of PROMs in quality evaluation, and, through the efficient use of resources, can help complex topics to be addressed. Both strategies can promote motivation and allay concerns. However, the task is ongoing and requires staff and time.

## Supplementary Information

**Additional file 1.**

## Data Availability

All online-survey data generated or analyzed during this study are included in this published article and its supplementary information files. As the focus group transcripts may provide enough information to allow participants to be identified, no original data will be published, but online supplemental material on data collection is provided.

## Abbreviations

CFIR: Consolidated Framework for Implementation Research
DRKS: German Clinical Trials Register (‚Deutsches Register Klinischer Studien‘)
EAPC: European Association for Palliative Care
EDS: Electronic documentation system
ELSAH: Evaluation of Specialised Outpatient Palliative Care by taking the example of Hesse
HP: Health professional
IPOS: Integrated Palliative Outcome Scale
IPOS VoC: IPOS Views on Care
NoMAD: Normalization MeAsure Development
NPT: Normalization Process Theory
OACC: Outcome Assessment and Complexity Collaborative
PCOC: Palliative Care Outcomes Collaboration
PROM: Patient reported outcome measure
SOPC: Specialized outpatient palliative care
MMARS: Mixed Methods Article Reporting Standards
ICMJE: International Committee of Medical Journal Editors
