# Supplementary material for: State-wide implementation of patient-reported outcome measures (PROMs) in specialized outpatient palliative care teams (ELSAH): A mixed-methods evaluation and implications for their sustainable ﻿use
Source: BMC Palliat Care. 2022 Dec 2;21:216. doi: 10.1186/s12904-022-01109-w (PMC9716659; doi:10.1186/s12904-022-01109-w)

Supplemental material

Article: State-wide implementation of patient-reported outcome measures in specialized outpatient palliative care teams (ELSAH): A mixed-methods evaluation and implications for their sustainable use

Authors: Hannah Seipp, Jörg Haasenritter, Michaela Hach, Dorothée Becker, Dania Schütze, Jennifer Engler, Stefan Bösner, Katrin Kuss

<u>Table of Contents:</u>

A. Mixed-Methods Article Reporting Standards (MMARS) 2

B. Focus group topic guide (translated into English) 4

C. Online-survey 5

C.1 General appraisal 5

C.2 Constructs of the NPT 6

C.2.1 Results in table 6

C.2.2 Results in bar chart 9

A. Mixed-Methods Article Reporting Standards (MMARS)

Based on:
Levitt HM, Bamberg M, Creswell JW, et al. Journal article reporting standards for qualitative primary, qualitative meta-analytic, and mixed-methods research in psychology: The APA Publications and Communications Board task force report. Am Psychol 2018; 73: 26–46.

Item	How this has been addressed
**Title page**	
Title	We have described the key issues and the study population. We have refrained from using qualitative or quantitative words, and referenced the term mixed-methods.
Author note	We report on funding, conflicts of interest, registration, affiliations, and provide contact information for the corresponding author. According to the Palliative Medicine Journal's instructions to authors, we provide some of this information on the title page and some in the declarations section.
**Abstract**	
Objectives	We frame the problem under investigation and state a clear research aim.
Participants	We describe the setting and participants in our study.
Study Method	We describe the mixed-methods design, including methods of data collection and triangulation.
Findings	We present the main results.
Conclusions	We present the major implications.
**Introduction**	
Description of research problems/ questions	We frame and synthesize relevant literature, and describe the problem of integrating outcome measures in daily care, the peculiarity of the setting of specialized palliative home-care, and the role of implementation frameworks and theory.
Study objectives/ aims/research goals	We provide the rationale behind using Normalization Process Theory. We describe the aim of the ELSAH study and the relation of this article to prior articles/publications. We explain the mixed-methods research aim of this article, which is the same as the aim of qualitative and quantitative methods.
**Method**	
Research design overview	We define the mixed-methods design, explain why mixed-methods are appropriate, and describe the focus groups and online-survey.
Participants or other data sources	We present the qualitative and quantitative data sources, and explain that our participants had the opportunity to use both methods. We present the methods in order of application. We provide the number of participants for each method.
Researcher description	We describe the researchers' experience in the methods section and their employment status in the declarations' section.
Participant recruitment	
Participant sampling or selection	We describe the invitation procedures for participation in the focus groups and online-survey in separate sections. We further describe cancelled participation.
Participant recruitment	We describe the setting and location, sampling method and invitation, and the informed consent procedures, as well as ethics approval and accordance with the Declaration of Helsinki.

Data collection	
Data collection/ identification procedures	We describe data collection strategies and discuss them in the strengths and limitations section. The focus group topic guide and the online-survey questions are available in the supplemental material. We mention the mean duration and duration range of the focus groups, and the duration of the online-survey.
Recording and transforming the data	We describe video-recording and transcription for the focus groups and the 'lime-survey' platform used in the online-survey.
Data analysis	
Validity, reliability, and methodological integrity	We describe the qualitative data analysis (including naming the coders, description of inductive/deductive coding, and the employed software), the quantitative data analysis (including the employed software), and the mixed-methods triangulation. We describe how the use of the triangulation protocol strengthens mixed-methods integrity.
**Findings/Results**	
Findings/Results subsections	We describe the qualitative and quantitative results, and how these results were integrated and mixed-methods results were derived.<br>We present the analytic process including quotes, and highlight the key results.
**Discussion**	
Discussion subsections	We discuss similarities and differences to previous theories and research findings, reflect on explanations of the findings, discuss strengths and limitations, and transferability, and derive implications of the integrated findings from use of the two methods.

B. Focus group topic guide (translated into English)

- **Please describe your general experience of using the measures.**
- **How do you currently rate their**
  - applicability?
  - comprehensibility?
  - usefulness in your practical work?
  - usefulness as a reflection of quality?
- **In your team, what do you do with the data you have collected using the measures?**
- **Has use of the outcome measures become standard procedure in your daily work?**
- **What could be improved?**
- **Do you have any feedback with respect to individual measures?**
- **Do you have any suggestions/wishes relating to the future use of the measures?**

C. Online-survey

C.1 General appraisal

Question	Response options	Answers n (%)	Mean value (SD; median)
What is your main role with respect to the 'standardized documentation of specialized palliative home-care in Hesse'?	I am responsible for the standardized documentation in my team.	19 (25)	-
	I use the standardized documentation in the day-to-day care of affected people.	49 (64.5)	-
	I use the standardized documentation as a member of the administrative team.	5 (6.6)	-
	Other	1 (1.3)	-
	n/a	2 (2.6)	-
Do you feel the modified documentation is an integral part of your day-to-day work?	Yes	52 (68.4)	-
	No	12 (15.8)	-
	n/a	12 (15.8)	-
If 'yes': To what extent is the modified documentation an integral part of your day-to-day work?	Scale 0 (not at all) - 10 (completely)	52	8,3 (2; 9)
If "No": Do you feel the modified documentation may become an integral part of your day-to-day work?	Scale 0 (not at all) - 10 (completely)	11	3,9 (2.5; 4)
	n/a	1	-
When you use the modified documentation, how familiar does it feel to you?	Scale 0 (not at all) - 10 (completely)	74 (97.4)	6,8 (2.5; 7)
	n/a	2 (2.6)	-
How often do you use elements of the standardized documentation during team meetings?	Regularly	24 (31.6)	-
	Frequently	10 (13.2)	-
	Occasionally	15 (19.7)	-
	Rarely	17 (22.4)	-
	Never	5 (6.6)	-
	n/a	5 (6.6)	-

n=76 participants; n/a = no answer; SD: standard deviation

C.2 Constructs of the NPT

C.2.1 Results in table

**Original question in German**	**English translation**	Strongly Agree	Agree	Neither agree nor disagree	Disagree	Strongly disagree	Not relevant to my role	No answer
**Coherence**								
Ich erkenne, wie sich die aktuelle Dokumentation von der bisherigen Art der Erfassung unterscheidet.	I can see how the current documentation differs from the previous method of collection.	35.53%	46.05%	11.84%	1.32%	1.32%	0.00%	3.95%
Die Mitarbeitenden meines Teams haben ein gemeinsames Verständnis des Ziels/Zwecks der veränderten Dokumentation.	Staff in my team have a common understanding of the purpose of the modified documentation.	9.21%	34.21%	17.11%	30.26%	6.58%	0.00%	2.63%
Ich erkenne den möglichen Mehrwert der veränderten Dokumentation für meine Arbeit.	I can see that the modified documentation has the potential to be useful in my work.	11.84%	25.00%	19.74%	21.05%	21.05%	1.32%	0.00%
Ich kann mich mit den Themen der veränderten Dokumentation identifizieren (bezieht sich auf den Inhalt, nicht auf die Formulierungen).	I can identify with the topics raised in the modified documentation (refers to content, not wording).	17.11%	39.47%	9.21%	19.74%	10.53%	1.32%	2.63%
Auf Basis der veränderten Dokumentation kann die Qualität der von meinem Team geleisteten Arbeit abgebildet werden.	The quality of the work performed in my team can be illustrated by using the modified documentation.	2.63%	23.68%	18.42%	32.89%	19.74%	1.32%	1.32%
Ich mache mir Gedanken, dass sich die veränderte Dokumentation nachteilig auf die SAPV auswirkt.	I am concerned that the modified documentation will have a negative impact on specialized palliative home-care.	14.47%	28.95%	25.00%	15.79%	7.89%	2.63%	5.26%
Zeit, die für Dokumentation aufgewendet wird, ist grundsätzlich sinnvoll investiert im Sinne der Versorgungsqualität/ Patientenversorgung.	Overall, time spent on documentation is invested wisely in terms of quality of care/patient care.	5.26%	25.00%	21.05%	26.32%	19.74%	1.32%	1.32%
**Cognitive participation**								
Es gibt Schlüsselpersonen in meinem Team, welche die kontinuierliche Anwendung der veränderten Dokumentation fördern.	There are key people in my team who encourage the continuous use of the modified documentation.	19.74%	42.11%	15.79%	7.89%	0.00%	3.95%	10.53%

Es gibt Schlüsselpersonen außerhalb meines Teams, welche die veränderte Dokumentation vorantreiben.	There are key people outside my team who encourage the use of the modified documentation.	11.84%	30.26%	15.79%	13.16%	5.26%	3.95%	19.74%
Ich weiß, wie die veränderte Dokumentation im Idealfall angewendet werden sollte.	I know how the modified documentation should ideally be used.	28.95%	53.95%	5.26%	2.63%	3.95%	1.32%	3.95%
Ich habe mich selbst oder mit anderen mit den Themen der veränderten Dokumentation auseinandergesetzt (theoretisch).	I have dealt with the topics raised in the modified documentation myself or with others (theoretically).	36.84%	48.68%	5.26%	3.95%	0.00%	1.32%	3.95%
Ich bin motiviert, die veränderte Dokumentation im Kontakt mit den Betroffenen im Alltag anzuwenden (praktisch).	I am keen to use the modified documentation in day-to-day contact with the persons concerned (practically).	13.16%	27.63%	15.79%	26.32%	11.84%	2.63%	2.63%
Ich befürworte die veränderte Dokumentation.	I am in favor of the modified documentation.	7.89%	21.05%	25.00%	25.00%	15.79%	1.32%	3.95%
**Collective action**								
Ich kann die veränderte Dokumentation problemlos in meine bestehende Arbeit integrieren.	I can easily integrate the modified documentation into my existing work.	3.95%	38.16%	18.42%	23.68%	7.89%	1.32%	6.58%
Die veränderte Dokumentation bringt Vorteile für die Zusammenarbeit mit Kollegen im Team.	The modified documentation benefits cooperation with colleagues in the team.	1.32%	17.11%	26.32%	28.95%	21.05%	1.32%	3.95%
Die veränderte Dokumentation bringt Vorteile für die Beziehungsqualität zu Patienten und Angehörigen.	The modified documentation benefits the quality of relationships with patients and relatives.	1.32%	11.84%	28.95%	27.63%	21.05%	3.95%	5.26%
Alle in meinem Team, die mit der veränderten Dokumentation im Alltag arbeiten müssen, können sie entsprechend anwenden.	Everyone in my team that has to work with the modified documentation in their day-to-day work can use it properly.	7.89%	63.16%	6.58%	14.47%	3.95%	0.00%	3.95%
Während der Einführung wurde genügend Unterstützung angeboten, damit die Mitarbeitenden die veränderte Dokumentation anwenden können.	Sufficient support was offered during the implementation phase to enable staff to use the modified documentation.	15.79%	47.37%	9.21%	15.79%	3.95%	0.00%	7.89%
Es wird aktuell genügend Unterstützung angeboten, damit die Mitarbeitenden die veränderte Dokumentation anwenden können.	Sufficient support is currently provided to enable staff to use the modified documentation.	11.84%	47.37%	17.11%	13.16%	2.63%	0.00%	7.89%
Es sind genügend Ressourcen vorhanden, um die veränderte Dokumentation im Alltag regelhaft anzuwenden.	Sufficient resources are available to ensure the modified documentation is used as intended in day-to-day care.	7.89%	31.58%	22.37%	22.37%	7.89%	1.32%	6.58%
Die Teamleitung unterstützt die veränderte Dokumentation hinreichend.	The team's management provides sufficient support for the modified documentation.	22.37%	50.00%	10.53%	1.32%	5.26%	2.63%	7.89%

**Reflexive monitoring**								
Ich kenne Berichte/Erfahrungen zur Nützlichkeit der veränderten Dokumentation.	I am aware of reports/experiences on the usefulness of the modified documentation.	2.63%	23.68%	23.68%	28.95%	14.47%	1.32%	5.26%
Ich schätze den Nutzen, den die veränderte Dokumentation auf meine eigene Arbeit hat.	I value the usefulness of the modified documentation in my own work.	1.32%	22.37%	23.68%	27.63%	19.74%	2.63%	2.63%
Meine Kollegen stimmen zu, dass die veränderte Dokumentation nützlich ist.	My colleagues agree that the modified documentation is useful.	0.00%	13.16%	22.37%	26.32%	25.00%	0.00%	13.16%
Ich habe Vertrauen, dass durch Feedback die veränderte Dokumentation künftig weiter verbessert wird.	I am confident that feedback will result in further improvements to the modified documentation in the future.	14.47%	44.74%	21.05%	10.53%	3.95%	1.32%	3.95%
Ich kann die Anwendung der veränderten Dokumentation an meine Arbeitsweise anpassen.	I can adapt the way I use the modified documentation to suit my way of working.	10.53%	50.00%	13.16%	11.84%	5.26%	3.95%	5.26%

C.2.2 Results in bar chart

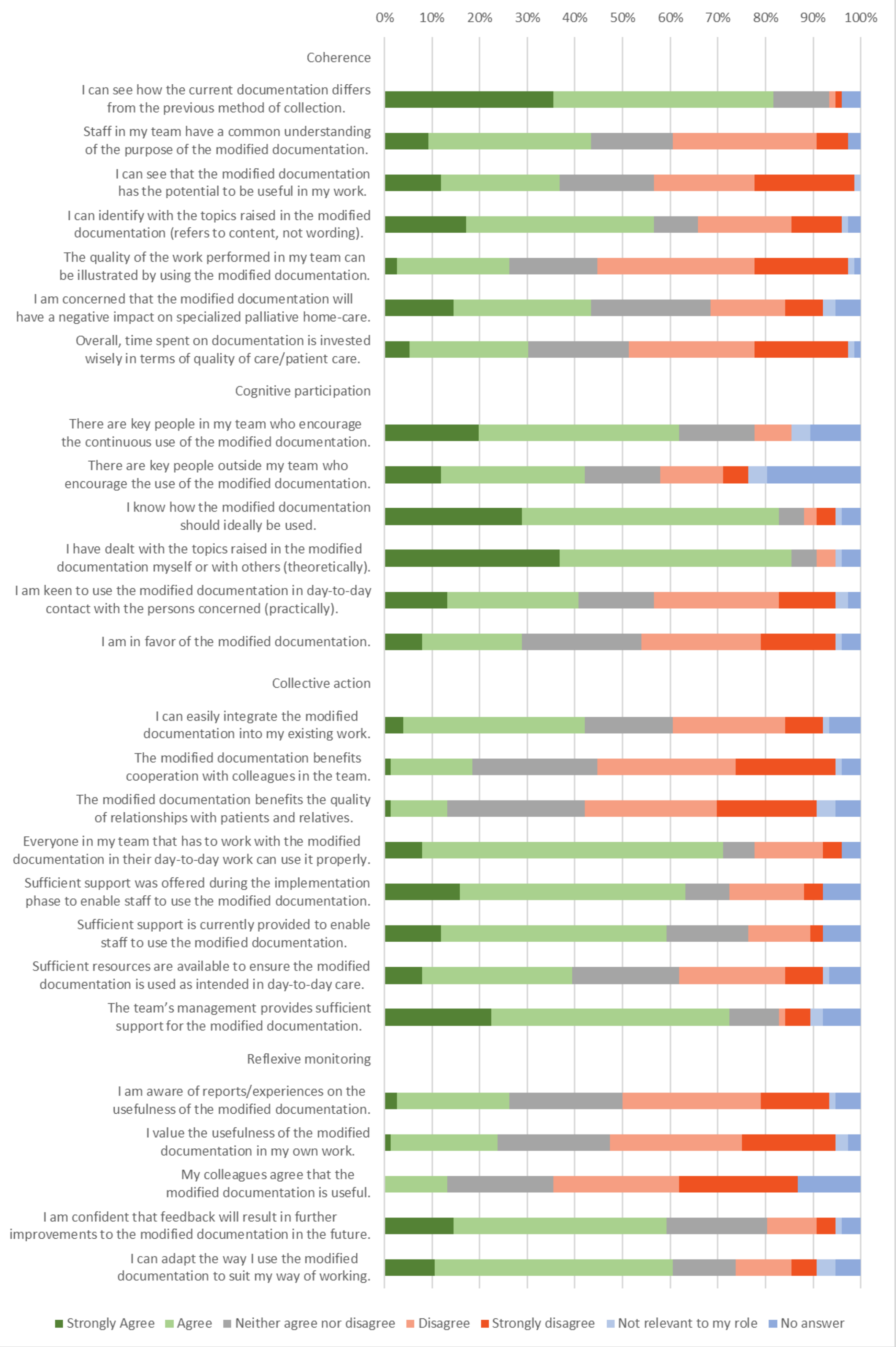

Supplement: Supplementary file 1 — Additional file 1. [file 12904_2022_1109_MOESM1_ESM.pdf]
